# Knowledge, Attitude and Practices of Postpartum Females Regarding the Acceptance of Immediate Postpartum Contraception: A Cross-Sectional Study From North India

**DOI:** 10.7759/cureus.29824

**Published:** 2022-10-02

**Authors:** Reena Pal, Sonam Maheshwari, Nirja Kaka, Neil Patel, Yashendra Sethi

**Affiliations:** 1 Department of Obstetrics and Gynaecology, Government Doon Medical College, Dehradun, IND; 2 Department of Community Medicine, Government Doon Medical College, Dehradun, IND; 3 Medicine and Surgery, GMERS Medical College, Himmatnagar, IND; 4 Department of Medicine, Government Doon Medical College, Dehradun, IND

**Keywords:** spacing, unplanned pregnancy, family planning, counseling, contraception, post-partum period

## Abstract

The immediate postpartum period is a great time to encourage the acceptance of contraceptive methods; the time is influenced by both emotional and physical factors. At this stage, the administration of intrauterine contraceptives is relatively easier with lesser complications due to the prior obstetric event. A single-center cross-sectional study was conducted using a self-constructed questionnaire-based interview on 331 women in their immediate postpartum period who had delivered a healthy live-born infant. The majority (59.8%) of study participants had unplanned pregnancies. We conducted behavior change communication sessions for postpartum family planning which resulted in 89% of participants accepting the methods with the prime reasons for acceptance being temporary child spacing (41%) and a definitive desire for no more children (34%). The odds were higher in women with more than five pregnancies [adjusted odds ratio (AOR) = 1.951, 95% CI = 1.389-2.925] and women whose last pregnancy was planned [AOR = 1.248, 95% CI = 1.002-3.215].The hindrance to adopt and adhere to postpartum contraception stems from a variety of socio-economic factors which are unique to low-income countries. Individually tailored behavior change communication/counseling approaches may help overcome misconceptions and meet the heterogeneous needs for family planning in the immediate postpartum phase.

## Introduction

Maternal mortality has always been a public health challenge. The global data suggest that 303,000 maternal deaths occur worldwide with 99% of them in developing countries [1]. Leading causes of maternal mortality include sepsis, hemorrhage, eclampsia/pre-eclampsia, obstructed labor, and complicated abortion. High-risk pregnancies and short inter-conception periods indirectly contribute to these complications [2]. High-risk pregnancies can be effectively managed by timely diagnosis and optimal treatment during pregnancy. It has been observed that preventing closely spaced pregnancies in both high-risk and normal pregnancies greatly reduces the risk for maternal complications like uterine rupture, premature birth, uteroplacental bleeding disorders, and eclampsia/pre-eclampsia, and fetal complications like autism, low birth weight, and congenital anomalies [3-5].

The pooled data from the Indian National Family Health Survey (NFHS- 5) has highlighted that the chances of infant and maternal survival would increase by 2.5 times with birth spacing of three to five years compared to that of less than two years [6,7]. Other researchers also found that spacing pregnancies more than two years apart can avert more than 30% of maternal mortalities and 10% of child mortality [8,9]. The largest proportion of women with an unmet need for contraception is those in their first year after childbirth [10]. The immediate postpartum period is a good opportunity for contraception counseling as women have an extended interaction with the reproductive healthcare system during this period. The same survey (NFHS-5) also found that the percentage of women of reproductive age using contraceptives has risen to 67% from 57% noted in the NFHS-4 indicating the impact of various programs and health education on patients. Ironically, Alum et al. observed that women with high income, low parity, history of contraception use, and an educated spouse were more likely to have an early resumption of sexual intercourse [11].

Postpartum family planning (PPFP) mainly focuses on the prevention of unintended and closed-spaced pregnancies through the first year following childbirth [12,13]. As per NHFS-5, India has around 9.4% of the target population (married women aged 15-49) who have an unmet need for family planning, with many states having higher percentages up to 27%. It has been observed that around three-fourths of the target population have a demand for family planning with 14% having a demand for spacing births while 63% for limiting births. The current unmet need for family planning in Uttarakhand is 8.8% [6]. To avoid the adverse outcomes associated with closely spaced births, medical guidelines recommend the implementation of a family planning method by six weeks postpartum. Protocols to implement these recommendations have been adopted around the world but have been unsuccessful [14]. Despite the felt need, the failure of current contraceptive guidelines and service delivery protocols, especially for young and low/null parity females in developing countries suggests a need for re-evaluation of knowledge, attitude, and practices among the target population [15].

Over the last two decades, about 130 million maternal deaths have occurred in India. Although we note a decline in national maternal mortality ratios (MMR), those from some poorer states of India still have high MMR [16]. Appropriate spacing methods and prevention of unplanned pregnancies is an unmet need in India deterring the country from achieving its MMR goals. Postpartum contraception can greatly help prevent maternal deaths due to closed-spaced pregnancies as the women are less likely to come back to the hospital for contraception. Identifying the reasons for denial and acceptance of immediate postpartum contraceptive methods is crucial to devising an approach to encourage the adoption of these methods. The planning for public health interventions depends on the reasoning and the regional variations; which due to the scarce data is difficult to generalize. The current study was designed to fill the gaps and contribute the data from Uttarakhand, North India, as the state has a higher MMR (192) than most states [16].

## Materials and methods

Study design, study setting, and study participants

A cross-sectional study was conducted at the Department of Obstetrics and Gynaecology in a tertiary care hospital in Uttarakhand on females in the immediate postpartum period admitted during the period July 2020 to June 2021.

Inclusion and exclusion criterion

The study included all women on day one of the postpartum period who had an uncomplicated vaginal delivery as these formed the best target group for counseling and behavior change communication. We ensured that the included women had no medical/surgical illness. We excluded the patients who had a complicated vaginal delivery or had a history of medical/surgical illness or who achieved pregnancy through artificial reproductive techniques like artificial insemination, or in vitro fertilization (IVF). Patients below the age of 18 were also excluded.

Sampling and sample size

We recruited 331 study participants by purposive sampling. The sample size was calculated using G Power statistical analysis software keeping Slope H1 at 0.05, slope H0 at 0, alpha error probability at 0.10,1-Beta error probability at 0.90, and standard deviation sigma-x and sigma-y as 0.

Consent and ethical approval

Informed written consent was taken from the participants, and a self-designed structured questionnaire was administered in local languages during the immediate (first day) postpartum period. The study was approved by the Institutional Ethical Committee, Government Doon Medical College with registration number IEC/GDMC/2020/87. 

Statistical analysis

Data were entered into Excel (Microsoft, Redmond, WA, USA) and analyzed using Statistical Package for Social Sciences (SPSS) version 28.0 (IBM Corp., Armonk, NY, USA). Qualitative data were described using numbers and percentages. Fisher's exact test and chi-square test were used to check the association. Risk for potential variables was carried out using adjusted logistic regression. Significance test results are quoted as two-tailed probabilities. 

## Results

Demographic profile

The age distribution of the sample was symmetrical around 25 years of age with half the population above and below it; the majority (68.6%) were Hindu, had literate partners (57.7%), and belonged to a service-class family with monthly income more than ₹20000. Around 63.1% of women got married between the age of 19-22 years. Most women in the study were para 1 (Table 1).

**Table 1:** Socio-demographic characteristics of the study participants (n = 331) * Service-teaching professionals, computer operators, and clerks ** Labor: Miners, construction workers, farmers

Variables	Frequency	Percentage
Age (years)		
18- 20	20	6.0
20-25	140	42.3
25+	171	51.7
Religion		
Hindu	227	68.6
Muslim	104	31.4
Maternal Education		
Illiterate	3	0.9
Secondary	40	12.1
High School	30	9.1
Intermediate	67	20.2
Graduation	158	47.7
Post-Graduation	33	10.0
Partner Education		
Illiterate	2	0.6
Secondary	6	1.8
High School	62	18.7
Intermediate	90	27.2
Graduation	106	32.0
Post-Graduation	65	19.6
Occupation		
House Worker	331	100.0
Partner Occupation		
Service*	209	63.1
Labor**	115	34.7
Others	7	2.1
Monthly Income		
Less than 10,000	85	25.7
10,000-20,000	98	29.6
20,000-25,000	148	44.7

Current status of knowledge and use of contraception

Among study participants, 59.8% had not planned the current pregnancy. However, a majority (79.8%) of them affirmed their knowledge about the available contraceptive methods. The most prevalent method amongst the participants were natural methods (abstinence, safe- period, lactational amenorrhoea) (78.3%), followed by the barrier methods (32.2%), oral contraceptive pills (6%), injectable depot medroxyprogesterone acetate, intrauterine contraceptive devices, and sterilization. Although most (79.8%) participants knew about contraception, less than half of them adopted family planning methods in the past (41%). 

The main reasons reported for not employing the contraceptive techniques were: plans for further childbearing, fear of infertility, lack of spousal support, and religious beliefs. The study participants who had not used any contraceptive method were counseled regarding postpartum family planning methods and their benefits. After counseling 89% of participants accepted to use any form of postpartum family planning methods. The main reasons for acceptance were temporary child spacing (41%) and the definitive desire for no more children (34%) (Table 2).

**Table 2:** Reproductive health and maternal health service-related characteristics of study participants

Variables	Frequency	Percentage
Parity		
1	209	63.1
2-3	109	32.9
4 or more	13	3.9
Age At Marriage		
≥18	9	2.7
19-22	203	61.3
22-25	109	32.9
More than 25	10	3.0
Duration of Marriage		
1-2	85	25.7
3-4	98	29.6
≥5	148	44.7
Previous Caesareans Sections		
0	224	67.7
1	107	32.3
≥2	0	0
Last Pregnancy		
Planned	133	40.2
Parity		
1	86	64.6
2-3	40	30
≥4	07	5.2
Unplanned	198	59.8
1	110	55.5
2-3	43	22
≥4	45	22.7

Among 89% of participants who had accepted the contraceptive methods most of them were primipara (65%). The most preferred methods in the primipara group were injectable contraceptives and intrauterine devices followed by the barrier and oral contraceptive pills. Among participants with two or three children, preferred methods were injectable contraceptives and intrauterine devices followed by sterilization and oral contraceptive pills. Participants with four or more children preferred intrauterine devices and sterilization as a family planning method. Eleven percent of participants did not opt for any contraceptive method due to their husbands’ dissent (6.6%) or were not able to decide which method will be good for them (5.5%) (Table 3). The most common reason couples avoided using contraception despite being aware was the desire for childbearing (18.7%) followed by fear of infertility (7.8%) (Table 4). 

**Table 3:** Descriptive statistics of the current contraceptive practices of the study participants IUD: intrauterine device, DMPA: depot medroxyprogesterone acetate, OCP: oral contraceptive pills

Variables	Frequency	Percentage
Having Knowledge of Contraception		
Yes	264	79.8
No	67	20.2
If yes, then which type		
Natural	136	51.5%
Natural / Barrier	78	29.5%
Natural / Barrier/ IUD	8	3%
Natural / Barrier/ IUD/ Oral Pills	4	4.5%
Natural / Barrier/ IUD/ Sterilization	12	3.8%
Natural / Barrier/ DMPA/ Oral pills	8	3%
Natural / IUD	10	3.8%
Natural / Barrier/ Oral pills	06	2.2%
IUD	2	2.2%
Previously Used		
Natural	259	78.3%
Natural / Barrier	6	18.3%
Barrier	46	13.9%
IUD	20	6%
Used Post-partum family planning methods		
Yes	108	41%
No	156	59%
Reason for acceptance after counseling (294)		
Definitive desire for no more children	101	34.3%
Child spacing, temporarily no children	121	41.2%
Satisfaction with prior contraceptives	72	24.5%
Preferred methods of contraception after counseling		
Parity - 1	190/205	
DMPA	65	34.2%
IUD	50	26.3%
Barrier	46	24.2%
OCP	29	15.3%
Parity –2-3	(95/109)	
DMPA	46	48.4%
IUD	30	31.7%
Sterilization	15	15.7%
OCP	4	4.2%
Parity 4 or more	(9/13)	
Sterilization	5	55.6%
IUD	3	33.3%
DMPA	1	11.1%
Reason for not acceptance after counseling		
Causes related to husband	22	6.6%
Not able to decide the method to choose	15	4.4%

**Table 4:** Reasons for not using contraception despite knowledge before present pregnancy

Reasons	(N=156)	(%)
Spousal refusal	24	15.38
Fear of infertility	26	16.67
Desire for further childbearing	62	39.74
Medical reasons	4	2.56
Religious belief	20	12.82
Side effects	11	7.05
No reason	9	5.77

Factors associated with contraceptive use during the postpartum period

A bivariate analysis was done for contraceptive use during the postpartum period. A Chi-square test was applied to test association and we report that maternal education, partner education, age at marriage, duration of the marriage, total pregnancies and previous cesarean sections, and last planned or unplanned pregnancy were all significantly associated with contraceptive use during the postpartum period. 

Contraceptive use during the postpartum period was influenced by many factors, we have analyzed the adjusted odds ratio for each factor and we found that the odds of accepting the contraceptive improved with education status: Compared to Illiterate women, postgraduate women had the highest odds of contraceptive use [adjusted odds ratio (AOR) = 2.061, 95% CI = 1.019-3.198]. Interestingly, women with educated partners had fewer odds of contraceptive use [AOR = 0.044, 95% CI = 0.005-0.384]. Women aged above 25 when married had higher odds of modern contraceptive use [AOR = 2.090, 95% CI = 1.429, 3.056] than women who married when younger, also; higher odds were noted for women whose duration of marriage was five or more years [AOR = 2.943, 95% CI = 1.579-3.990]. Women with more pregnancies had higher odds than women with one to two pregnancies. Women whose last pregnancy was planned had higher odds [AOR = 1.248, 95% CI = 1.002-3.215] relative to those who had an unplanned pregnancy (Table 5).

**Table 5:** Adjusted odds ratios (AOR) and 95% confidence intervals (CI) of factors associated with contraceptive use during the postpartum period (n = 331) *p<0.05 PPFP: postpartum family planning

Variables	Category	PPFP Used(294)	PPFP not Used(37)	Chi Square, p- value	AOR (95% CI)
Age	≥20	16	4	2.27, 0.321	1
	21-25	123	17		1.030(0.396-2.680)
	25+	155	16		1.258(0.490-3.233)
Religion	Hindu	201	26	0.055,0.814	1.002(0.9455-2.759)
	Muslim	93	11		1
Maternal Education	Illiterate/ Secondary	40	3	12.01, 0.016	1
	High School	21	9		1.129(0.380-3.355)
	Intermediate	60	7		1.692(1.128-2.683)*
	Graduation	143	15		1.169(1.079-2.361)*
	Post-Graduation	30	3		2.061(1.019-3.198)*
Partner Education	Illiterate/ Secondary	6	2	111.42,0.000	
	High School	32	30		1.260(0.030-2.250)
	Intermediate	88	2		0.286(0.034-2.430)
	Graduation	106	0		0.035(0.004-0.299)*
	Post-Graduation	62	3		0.044(0.005-0.384)*
Age At Marriage	≥18	5	4	19.69,0.000	1
	19-22	183	20		1.537(1.068-2.211)*
	22-25	100	9		1.681(1.144-2.470)*
	More than 25	6	4		2.090(1.429-3.056)*
Duration of Marriage	1-2	67	18	11.55, 0.003	1
	3-4	90	8		1.256(0.958-2.071)
	≥5	137	11		2.943(1.579-3.990)*
Total Pregnancies	1-2	195	14	11.72, 0.002	1
	3-4	89	20		1.366(0.158-1.771)
	≥5	10	3		1.951(1.389-2.925)*
Previous Caesareans Sections	0	244	23	9.14, 0.002	1
	1	50	14		0.245(0.087-0.874)*
Last Pregnancy	Planned	110	23	8.37, 0.000	1.248(1.002-3.215)*
	Unplanned	184	14		

## Discussion

Maternal morbidity and mortality present a big challenge globally with the lower-middle income countries facing a higher burden due to the short inter-conception period. Early initiation of sexual activity, poor breastfeeding, and aversion to contraception can pose a risk of unintended closely spaced pregnancy. Postpartum family planning counseling can reduce both maternal as well as neonatal morbidity and mortality [17,18]. Breastfeeding confers natural protection to pregnancy for the first six months postpartum due to prolactin-induced anovulation and amenorrhea. Thus, lactational amenorrhea (LAM) is a natural, safe, and easy-to-adopt contraceptive. Although easy to implement, it is not very effective since it requires strict adherence to breastfeeding without top feeding. Despite the shortcomings of this method, women should be counseled and offered the choice to opt for it as breastfeeding also offers protective benefits to infants [19].

Knowledge of contraceptive methods

The proportion of subjects with appropriate knowledge about contraception in our study (79.8%) was similar to that of studies conducted in Liberia (79%) and Indonesia (77%) [20,21]. Despite having appropriate knowledge about the benefits and methods of contraception, Joshi et al. found it was used much lesser than required in Nepal, another developing country like India [21]. We attribute this inadequate usage to the socio-demographic structure, educational status, awareness, and availability of services in countries with low socio-economic status.

Attitude and practices towards immediate postpartum contraception

Despite the knowledge of contraception, less than half (41%) of the study population used contraceptives previously and for more than half of the participants (59.8%), the current pregnancy was unplanned. Counseling and interactive sessions helped these couples adopt contraceptive techniques and promoted adherence amongst users. Hesitation to adopt and non-adherence despite availability and awareness indicates that there are unaddressed reservations in this population that need to be identified and quelled [21].

Factors influencing the resistance to contraceptives

The reservations participants had with using contraceptives stemmed from unaddressed concerns: fear of permanent infertility (7.8%), lack of spousal support (7.2%), and religious belief (6.6%). Sachdeva et al. and Kumar et al. recognized that refusal to use contraceptives was also due to irregular menstruation and weakness [22,23]. We noted that both spouses had a say in whether they used contraception, which resonates with the existing literature [23,24]. The authors were alarmed to find that a majority of married women in India still endure the male marital hierarchy and do not make independent contraceptive decisions, even in urban settings.

Postpartum contraception

We found that the participants are most open to accepting the use of contraception immediately postpartum and are open to family planning counsel. The need for family planning is often unmet, and the stigma associated with its usage deepens the grave [20,25,26]. But many other studies report that only half of the postpartum contraception non-users intended to use any form of contraception in the future [27,28].

Interestingly, women with educated partners had fewer odds of contraceptive use which indicates that the decision-making is influenced by socio-cultural factors and joint decision-making honoring the advice of their parents. This contrasts the findings of studies done in Jimma, North West Ethiopia, and other parts of rural India, thus underlining the challenges arising from regional cultural changes and education status [29-31]. The role of the family in contraceptive use cannot be ignored as the closely related people influence decision-making. Smith et al. highlighted that the knowledge and attitude of postpartum mothers to family planning methods were associated with both partners' educational status, we expand that to include the role of families and culture as well [32]. As expected, the attitude of most postpartum mothers (89%) towards contraception was positive, hence focused counseling would be pivotal at this stage. Semachew Kasa et al. had similar findings in Western Ethiopia [33]. 

The preferred methods of contraception varied with parity: primiparous women preferred injectable contraceptives and intrauterine devices followed by barrier contraceptives and oral contraceptive pills; multiparous women preferred injectables and sterilization. Our finding conforms well with the results for India in the world contraceptive use pattern survey [34-36]. There is no baseline data on the proportion of postpartum women attending further scheduled contraceptive clinic visits. More importantly, the reasons for the poor turnover are also not determined. This information is vital to planning interventions aiming to increase contraceptive acceptance among postpartum women.

Proper understanding and effective implementation of behavior change communication strategies in the immediate postpartum period as done in our study can prove highly effective in promoting contraceptive acceptance and reducing the burden of maternal mortality and morbidity arising from an unmet need for contraception in low-income countries.

Limitations

We recognize our study is a single-center cross-sectional study and hence generalizability of findings to the entire region is not possible. Also, the data derived during the postpartum phase may be influenced by a catena of emotional factors which might influence the results.

## Conclusions

The hindrance to adopting and adhering to postpartum contraception stems from a variety of socio-economic factors which are unique to low-income countries. Timely and tailored behavior change communication/counseling approaches may help overcome misconceptions and meet the heterogeneous needs for family planning in the postpartum phase.

## References

[1] Damtie Y, Kefale B, Yalew M, Arefaynie M, Adane B (2021). Short birth spacing and its association with maternal educational status, contraceptive use, and duration of breastfeeding in Ethiopia. A systematic review and meta-analysis. PLoS One.

[2] Collier AY, Molina RL (2019). Maternal mortality in the united states: updates on trends, causes, and solutions. Neoreviews.

[3] Conde-Agudelo A, Rosas-Bermúdez A, Kafury-Goeta AC (2007). Effects of birth spacing on maternal health: a systematic review. Am J Obstet Gynecol.

[4] Cheslack-Postava K, Liu K, Bearman PS (2011). Closely spaced pregnancies are associated with increased odds of autism in California sibling births. Pediatrics.

[5] Grisaru-Granovsky S, Gordon ES, Haklai Z, Samueloff A, Schimmel MM (2009). Effect of interpregnancy interval on adverse perinatal outcomes--a national study. Contraception.

[6] (2021). India National Family Health Survey (NFHS-5). http://rchiips.org/nfhs/.

[7] Shah IH, Say L (2007). Maternal mortality and maternity care from 1990 to 2005: uneven but important gains. Reprod Health Matters.

[8] Abdul-Hamid I (2018). Abdul-Hamid I. Adolescents’ Knowledge, Attitudes and Perceptions Regarding Sexual and Reproductive Health and Teenage Pregnancy in LA, Greater Accra Region.

[9] Cleland J, Bernstein S, Ezeh A, Faundes A, Glasier A, Innis J (2006). Family planning: the unfinished agenda. Lancet.

[10] Ross JA, Winfrey WL (2001). Contraceptive use, intention to use and unmet need during the extended postpartum period. Int Fam Plan Perspect.

[11] Alum AC, Kizza IB, Osingada CP, Katende G, Kaye DK (2015). Factors associated with early resumption of sexual intercourse among postnatal women in Uganda. Reprod Health.

[12] DaVanzo J, Hale L, Razzaque A, Rahman M (2007). Effects of interpregnancy interval and outcome of the preceding pregnancy on pregnancy outcomes in Matlab, Bangladesh. BJOG.

[13] Rutstein SO (2005). Effects of preceding birth intervals on neonatal, infant and under-five years mortality and nutritional status in developing countries: evidence from the demographic and health surveys. Int J Gynaecol Obstet.

[14] world health o (2013). Programming Strategies for Postpartum Family Planning. https://apps.who.int/iris/bitstream/handle/10665/93680/9789241506496_eng.pdf.

[15] Jansen WH 2nd (2005). Existing demand for birth spacing in developing countries: perspectives from household survey data. Int J Gynaecol Obstet.

[16] Meh C, Sharma A, Ram U (2022). Trends in maternal mortality in India over two decades in nationally representative surveys. BJOG.

[17] Singh S, Sedgh G, Hussain R (2010). Unintended pregnancy: worldwide levels, trends, and outcomes. Stud Fam Plann.

[18] Hubacher D, Mavranezouli I, McGinn E (2008). Unintended pregnancy in sub-Saharan Africa: magnitude of the problem and potential role of contraceptive implants to alleviate it. Contraception.

[19] Van der Wijden C, Manion C (2015). Lactational amenorrhoea method for family planning. Cochrane Database Syst Rev.

[20] Kaydor VK, Adeoye IA, Olowolafe TA, Adekunle AO (2018). Barriers to acceptance of post-partum family planning among women in Montserrado County, Liberia. Niger Postgrad Med J.

[21] Joshi AK, Tiwari DP, Poudyal A, Shrestha N, Acharya U, Dhungana GP (2020). Utilization of family planning methods among postpartum mothers in Kailali District, Nepal. Int J Womens Health.

[22] Sachdeva A, Gupta A, Kumar D, Singh H, Sharma S (2017). Unmet need for family planning among married women of reproductive age group in rural and urban area of Shimla, India. Int J Med Sci Public Health.

[23] Kumar S, Priyadarshni A, Kant S, Anand K, Yadav BK (2005). Attitude of women towards family planning methods and its use--study from a slum of Delhi. Kathmandu Univ Med J (KUMJ).

[24] Idowu A, Deji SA, Ogunlaja O, Olajide SO (2015). Determinants of intention to use post partum family planning among women attending immunization clinic of a tertiary hospital in Nigeria. Am J Public Health Res.

[25] Jalang'o R, Thuita F, Barasa SO, Njoroge P (2017). Determinants of contraceptive use among postpartum women in a county hospital in rural Kenya. BMC Public Health.

[26] Thapa K, Dhital R, Rajbhandari S (2020). Prevalence of postpartum family planning service coverage in selected referral facilities of Nepal. JNMA J Nepal Med Assoc.

[27] Bajracharya A (2015). Knowledge, attitude and practice of contraception among postpartum women attending Kathmandu Medical College Teaching Hospital. Kathmandu Univ Med J (KUMJ).

[28] Mengesha ZB, Worku AG, Feleke SA (2015). Contraceptive adoption in the extended postpartum period is low in Northwest Ethiopia. BMC Pregnancy Childbirth.

[29] Haile A, Enqueselassie F (2009). Influence of women's autonomy on couple's contraception use in Jimma town, Ethiopia. Ethiop J Health Dev.

[30] Shah NJ, Pradhan P, Reddy AS, Joseph B (2006). Contraceptive practices in newly married women in sub-urban Bangalore. Health Popul Perspect Issues.

[31] Reed E, Donta B, Dasgupta A (2016). Access to money and relation to women’s use of family planning methods among young married women in rural India. Matern Child Health J.

[32] Smith E, Charantimath US, Wilson SF, Hoffman MK (2017). Family planning in Southern India: a survey of women's attitudes. Health Care Women Int.

[33] Semachew Kasa A, Tarekegn M, Embiale N (2018). Knowledge, attitude and practice towards family planning among reproductive age women in a resource limited settings of Northwest Ethiopia. BMC Res Notes.

[34] Alemayehu M, Lemma H, Abrha K (2016). Family planning use and associated factors among pastoralist community of Afar region, eastern Ethiopia. BMC Womens Health.

[35] Goel S, Bhatnagar I, Khan ME (2010). Increasing postpartum contraception in rural Uttar Pradesh. J Fam Welf.

[36] Kashyap C, Mohanty IR, Thamke P, Deshmukh YA (2017). Acceptance of contraceptive methods among postpartum women in a tertiary care center. J Obstet Gynaecol India.

